# Mesoporous Metal–Organic Framework from Templated Synthesis as Mechanical Metamaterials

**DOI:** 10.1021/jacs.5c04214

**Published:** 2025-06-29

**Authors:** Ting-Wei Liang, Chien Chen, Shinpei Kusaka, Suhail K. Siddique, Cheng-Yen Chang, Ryotaro Matsuda, Rong-Ming Ho

**Affiliations:** † Department of Chemical Engineering, 34881 National Tsing Hua University No. 101, Section 2, Kuang-Fu Road, Hsinchu 30013, Taiwan, R.O.C; ‡ Department of Materials Chemistry, Graduate School of Engineering, 12965 Nagoya University, Furo-cyo, Chikusa-ku, Nagoya 464-8603, Japan; § Department of Nuclear and Mechanical Engineering, Khalifa University of Science and Technology, Abu Dhabi 127788, UAE; # Integrated Research Consortium on Chemical Science (IRCCS), Furo-cho, Chikusa-ku, Nagoya 464-8602, Japan

## Abstract

This work aims to demonstrate the fabrication of a mesoporous metal–organic framework (MOF) via templated synthesis using a self-assembled block copolymer as a template, giving a nanonetwork MOF with enhanced toughness due to the effect of deliberate structuring on mechanical performance (the character of mechanical metamaterials). Polystyrene-*b*-polydimethylsiloxane (PS-*b*-PDMS) can self-assemble as a diamond phase, followed by hydrofluoric acid etching of PDMS, giving mesoporous PS as a template for the coordination-driven self-assembly reaction of ZIF-67. After the templated synthesis of ZIF-67, followed by removal of the PS template, diamond-structured mesoporous ZIF-67 with faceted texture can be obtained due to the confined growth of ZIF-67 as a single crystal. The deliberate structuring with the nanonetwork struts of the mesoporous ZIF-67 single crystal gives a significant improvement in energy dissipation capability with a brittle-to-ductile transition, as evidenced by nanoindentation tests, offering promising catalytic applications by improving the accessible active sites in mesoporous MOF and material toughness due to the nanonetwork structure.

## Graphical abstract

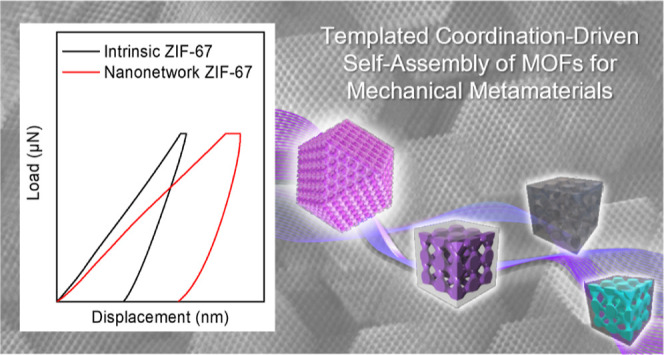

## Introduction

The creation and structuring of materials with precise, well-defined architectures, especially at micro- and nanoscale levels, have garnered considerable interest due to their remarkable mechanical attributes, including lightweight, high specific strength, and superior energy dissipation.^1–3^ Achieving these properties necessitates advanced fabrication techniques that enable the production of ordered materials across various length scales.^4^ Despite the progress in 3D fabrication methods like additive manufacturing for microcellular materials,^5^ the top-down approaches face limitations in nanoscale material options and are constrained by low throughput due to the extensive time and energy required.^6,7^

An emergent approach involves the self-assembly of block copolymers (BCPs), which allows for the creation of nanostructured materials with meticulous control over their dimensions and shapes by adjusting the composition and molecular weights of the BCPs.^8–10^ This process can generate nanoporous polymeric templates with well-defined nanochannels through the selective removal of one constituted polymer component. By infiltrating these nanoporous templates with suitable precursors, solidifying them into a rigid framework, and subsequently removing the template, periodic 3D nanostructured materials can be produced.^11–13^ Recent studies have extensively utilized self-assembled BCPs as templates to synthesize a variety of well-defined nanostructured materials, including thermoset resins,^14^ metals,^15^ and metal oxides.^16^ Among the array of periodic nanostructures derived from BCP self-assembly, well-ordered nanonetwork architectures like gyroid^17,18^ and diamond^19^ textures stand out due to their unique nanoscale geometries, offering properties that surpass those found in nature. Drawing inspiration from natural structural principles, extensive studies focus on developing porous materials with ordered architectures that aim to improve the mechanical properties of the material.^2,20,21^ It has also been proven that hierarchical porosity can greatly enhance the accessibility of active sites and facilitate better mass diffusion.^22,23^

This approach combines the structural benefits of nanonetwork geometries with the functional advantages of increased porosity, thereby creating materials with substantial potential for advanced applications in catalysis, filtration, and energy storage.^4,24^ These materials with superior performance are promising due to their optimized mechanical and functional properties.

Metal–organic frameworks (MOFs)^25^ are crystalline materials distinguished by their highly ordered structures and tunable properties, making them unique among porous solids. Unlike many traditional porous materials, such as activated carbons^26^ and molecular sieves,^27,28^ which typically exhibit disordered and undefined structures, MOFs offer a high degree of control over their pore sizes and the distribution of functional sites within their frameworks.^29–31^ This precision enables the design of MOFs with tailored chemical functionality and structural characteristics, making them highly versatile. MOFs are composed of metal ions or clusters coordinated to organic ligands, which create a vast array of possible structures. This variability allows for the engineering of MOFs with specific pore environments and functionalities that are well-suited for a variety of applications. Their exceptionally high surface areas, surpassing those of many zeolites,^32^ along with their structural diversity, make MOFs highly effective for gas storage and separation, catalysis, environmental remediation, sensing, and even pharmaceutical delivery. The ability to finely tune the properties of MOFs offers significant advantages for researchers and industries seeking to develop new materials with optimized performance for specific applications.

Recently, a comprehensive review on hierarchical porous MOFs and templating strategies highlighted the feasibility in enhancing mass transport and increasing surface accessibility for catalysis and separation from the hierarchical MOF structures via template-based synthesis.^33,34^ Significant advancements in terms of applications such as catalysis,^35^ biomedical functions,^36^ sensing,^37^ and separation^38^ have been achieved due to their deliberate structure fabrication.

By taking advantage of the hierarchically porous MOFs fabricated, it is feasible to provide advanced properties in a variety of applications. For instance, owing to their deliberate structure fabrication, 2D flexible MOFs can be fabricated, giving origami-inspired, auxetic behavior enabled by reversible folding and unfolding of the framework.^39^ Also, hollow UiO-66 crystals with hierarchical porosity can be fabricated, giving high strength with ultralight MOF-based mechanical metamaterials, due to framing.^40^ As mentioned above, this work aims to emphasize the enhancement of mechanical performance resulting from the effect of deliberate structuring on mechanical properties (the characteristic of mechanical metamaterials) through templated synthesis using self-assembled BCPs as templates for the fabrication of well-ordered mesoporous MOF frameworks with faceted single-crystal ZIF-67 due to the special growth mechanism from templated coordination-driven crystallization, giving new insights into the templating strategies of MOFs and the corresponding hierarchical porous MOFs fabricated for the applications that require superior mechanical properties such as toughness and energy dissipation capability. Beyond its mechanical properties, the introduction of mesopores is expected to enhance mass transport and improve the accessibility of active sites, which are key factors for potential catalytic applications. The interconnected mesoporous framework allows guest molecules to move more freely within the material, helping to overcome diffusion limitations that are typical of purely microporous MOFs, particularly when larger molecules are involved.

As illustrated in Figure 1a, diamond-structured PS-*b*-PDMS can be obtained from solution-casting of PS-*b*-PDMS.^13,41,42^ Followed by hydrofluoric acid etching of PDMS, mesoporous PS with well-ordered (diamond-structured) nanochannels can be formed and then used as a template (Figure 1b).^43^ PS/ZIF-67 nanohybrids can be fabricated by a templated coordination-driven assembly reaction of Co­(NO_3_)_2_·6H_2_O and 2-methylimidazole (metal cluster and organic linker, respectively) (Figure 1c). After the removal of the PS template, diamond-structured mesoporous ZIF-67 can be fabricated (Figure 1d). Note that, owing to the aimed reaction through nucleation and growth mechanism, the mesoporous ZIF-67 fabricated gives the formation of particle texture, with sizes ranging from hundreds of nanometers to 10 μm. With the confined growth of mesoporous ZIF-67 within the template, a single-crystalline ZIF-67 can be obtained, thus giving a particle texture with faceted morphology as illustrated. The deliberate structuring of the mesoporous ZIF-67 single crystal gives significant improvement on energy dissipation capability with a brittle-to-ductile transition, as evidenced by nanoindentation tests. Also, previous studies have demonstrated that BCPs can serve as effective structure-directing agents in MOF systems. For instance, polymer–MOF hybrids were synthesized through covalent or physical incorporation of BCPs, enabling control over particle shape, crystal habit, and spatial organization.^44^ Similarly, the use of amphiphilic BCPs as soft templates has allowed the creation of mesoporous metal-based materials with tunable morphology and functionality for catalysis and sensing.^45^

**Figure 1:**
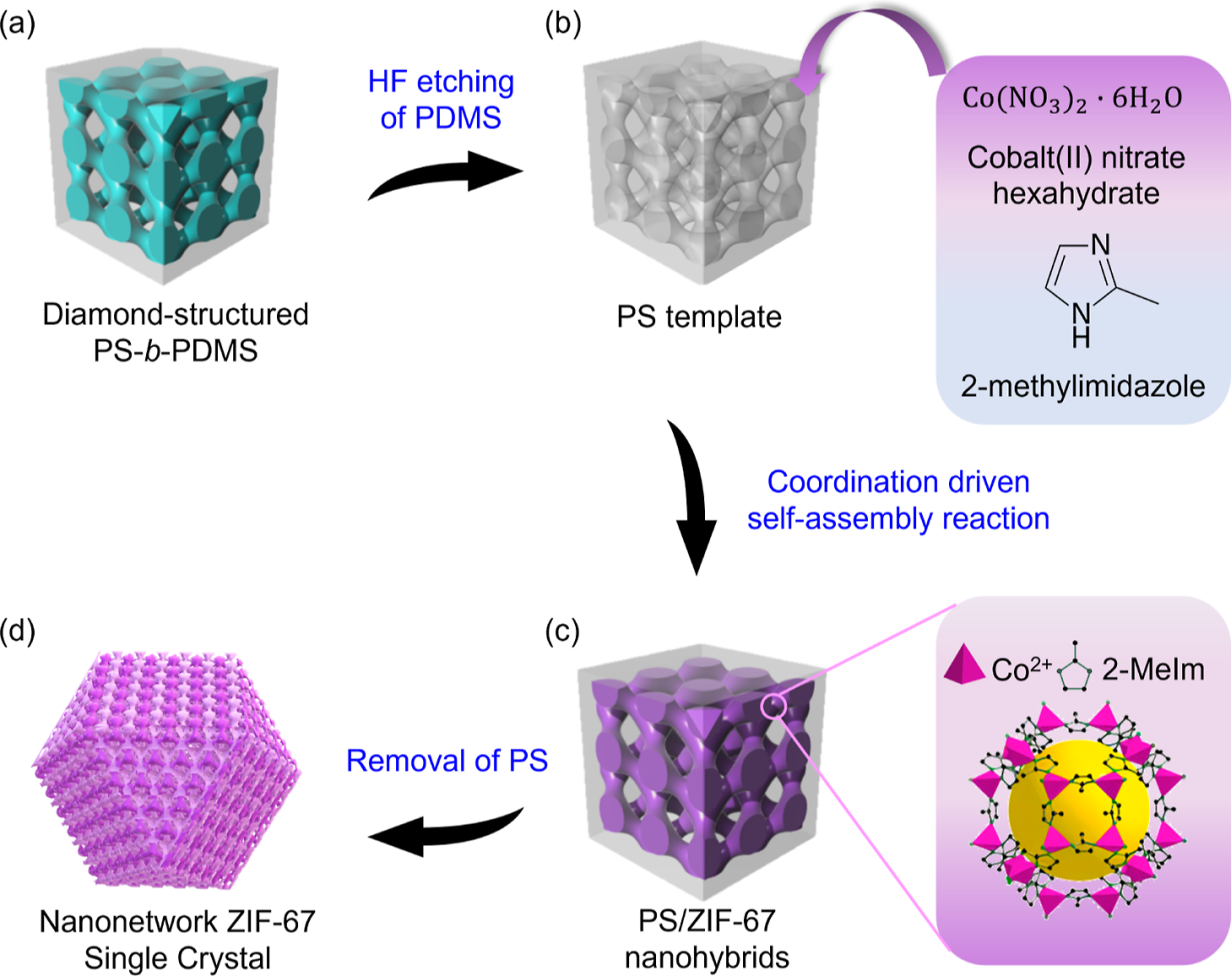
Schematic illustration for the fabrication of diamond-structured ZIF-67 single crystal from a templated coordination-driven self-assembly reaction using self-assembled PS-*b*-PDMS as a template: (a) diamond-structured PS-*b*-PDMS fabricated from solution casting of lamellae-forming PS-*b*-PDMS using chloroform as a PS-selective solvent; (b) mesoporous PS with well-defined (diamond-structured) nanochannels fabricated after hydrofluoric acid etching of the self-assembled PS-*b*-PDMS that can be used as a template; (c) pore filling of ZIF-67 precursors (Co­(NO_3_)_2_·6H_2_O and 2-methylimidazole) for templated coordination-driven self-assembly reaction; (d) diamond-structured ZIF-67 single crystal with faceted texture after the removal of PS for the PS/ZIF-67 nanohybrids fabricated.

## Results and Discussion

Owing to the high interaction parameter between PS and PDMS, self-assembled PS-*b*-PDMS with a diamond phase can be successfully obtained through solution casting of lamellae-forming PS-*b*-PDMS using chloroform as a PS-selective solvent.^41,42^ Figure S1 shows that when cyclohexane (a neutral solvent) was used, a lamellar structure will be formed, as evidenced by TEM and small-angle X-ray scattering (SAXS) results. Thermal annealing further enhances the structural order. Figure 2a displays the TEM projection of double diamond-structured PS-*b*-PDMS along the [311] direction obtained through solution casting using chloroform, a PS-selective solvent. The corresponding 1D SAXS profile (Figure 2b­(i)) reveals the reflections at the relative *q* values of $\sqrt{2},\sqrt{3},\sqrt{4}$ , $\sqrt{6}$ , $\sqrt{8}$ , $\sqrt{10},\sqrt{14}$ , and $\sqrt{22}$ , further confirming the formation of double diamond-structured PS-*b*-PDMS from self-assembly. The additional weak reflection at the low *q* region is attributed to the triclinic variant and/or the uniaxial contraction during the solution-casting process.^46^ As shown in Figure 2b­(ii), after the removal of PDMS by hydrofluoric acid etching, the 1D SAXS profile of the mesoporous PS template exhibits no significant changes in the relative *q* values between the solution-cast PS-*b*-PDMS and the mesoporous PS fabricated. Consequently, a mesoporous PS with diamond-structured nanochannels can be prepared and used as a template for the coordination-driven self-assembly reaction of MOFs.

**Figure 2:**
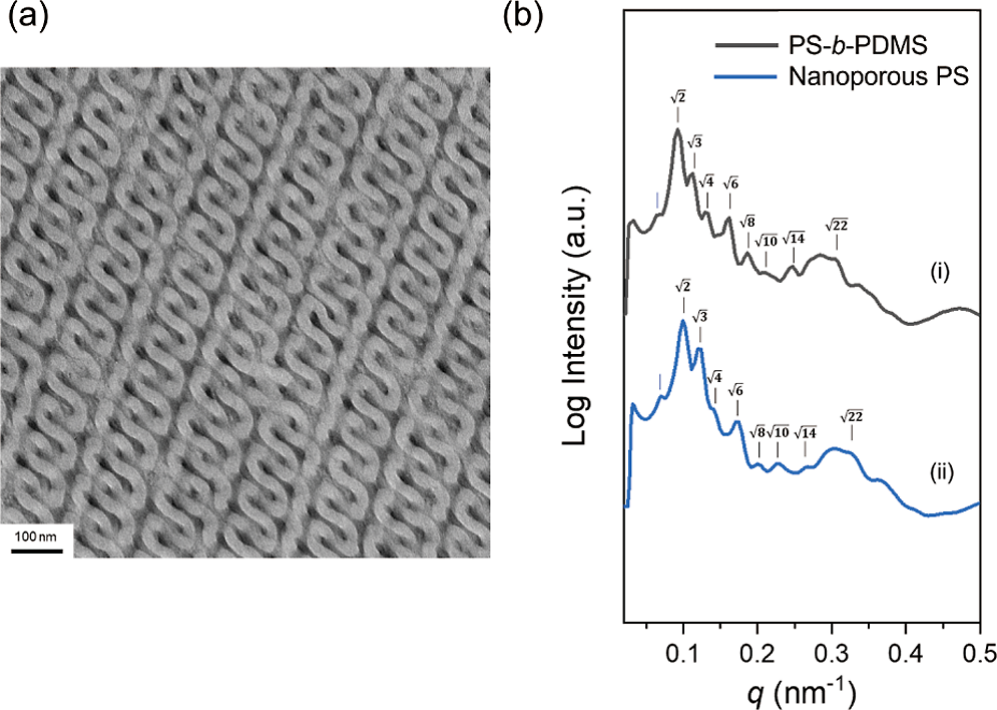
(a) TEM micrograph of PS-*b*-PDMS from solution casting with the use of chloroform as a PS-selective solvent. (b) 1D SAXS profiles of (i) solution-cast PS-*b*-PDMS; (ii) mesoporous PS after the removal of PDMS by hydrofluoric acid etching of (i).

The coordination-driven self-assembly reaction of ZIF-67 was carried out using a conventional solvothermal method in which DMF, H_2_O, and methanol can be used as guest molecules for the synthesis of ZIF-67 from the precursors of Co­(NO_3_)_2_·6H_2_O and 2-methylimidazole (metal cluster and organic linker, respectively). Note that the PS template fabricated is soluble in DMF, whereas the hydrophobicity of the PS template might inhibit the pore filling of an aqueous solution. Accordingly, methanol was chosen as a guest molecule for the templated synthesis of ZIF-67, while it can also serve as a surfactant for the aimed pore filling. Accordingly, Co­(NO_3_)_2_·6H_2_O and 2-methylimidazole were dissolved in a methanol solvent, respectively. To prevent rapid reactions of the precursors, which can block nanochannels by forming large nanocrystals, and thus hinder pore filling, the precursor solution was impregnated into the template one after another. The mesoporous PS template was first soaked in the metal ion precursor solution. Next, the fully impregnated template was transferred to the organic ligand precursor solution to ensure that the coordination-driven self-assembly reaction mostly occurs within the nanochannels of the PS template. Note that the stoichiometry of the organic linker to metal cluster for the formation of ZIF-67 is typically 2:1. However, for a successful coordination-driven self-assembly reaction, the ratio should be increased to 8:1 to ensure the completion of the desired reaction.^47^ Figure 3a shows the TEM micrograph of PS/ZIF-67 nanohybrids fabricated by the templated synthesis without staining. It can be observed that the double diamond projection is along the [111] direction at which the minor phase of the diamond structure shows a darker contrast resulting from the presence of cobalt ions in ZIF-67, confirming the successful pore-filling process, followed by the coordination-driven self-assembly reaction of ZIF-67. The field-emission scanning electron microscopy (FESEM) image of mesoporous ZIF-67 (Figure 3b) reveals a well-defined cuboctahedron texture with a uniform particle size distribution, achieved after the PS template was removed using chloroform. As a result, the diamond structure of the polymer template can be well preserved after the removal of the template. To further examine the morphology and the corresponding crystallographic structure, we further characterized the mesoporous ZIF-67 fabricated by SAXS and wide-angle X-ray diffraction (WAXD) simultaneously. As shown in Figure 4a, a well-defined 2D SAXS pattern with single-crystal-like reflections can be obtained from the mesoporous ZIF-67 fabricated. The corresponding 1D SAXS profile with characteristic reflections that occurred at the relative *q* values of $\sqrt{3},\sqrt{8},\sqrt{12}$ , $\sqrt{16}$ , $\sqrt{19}$ , $\sqrt{27}$ suggests the formation of single diamond structure (*Fd*3̅*m*);^48^ accordingly, the 2D SAXS pattern can be well indexed as shown. Note that, owing to the nucleation density-dependent growth mechanism in the coordination-driven self-assembly of ZIF-67 within the BCP template, only one of the two interpenetrating diamond-structured nanochannels is initiated for further growth. Once nucleation occurs in one domain, the subsequent growth proceeds preferentially along that network, while the other remains unoccupied due to spatial competition and precursor depletion. Consequently, a single diamond-structured ZIF-67 is formed even when using a double diamond phase as the template.^49^ Also, the 2D WAXD pattern with recognized reflections suggests that the fabricated diamond-structured ZIF-67 possesses high crystallinity (Figure 4b). The corresponding 1D X-ray scattering profile (Figure 4c) can be obtained by azimuthally integrating the 2D X-ray diffraction pattern. Sharp and strong XRD peaks with the reflections of (011), (002), (112), (022), (013), (222), (321), (330), (233), (422), (134), (521), (440), (530), and (600) corresponding to the crystallographic planes of ZIF-67 with the space group of *I*4̅3*m* can be clearly identified, further evidenced by the formation of ZIF-67 with high crystallinity from the templated synthesis. Owing to the growth size of the ZIF-67 crystals from the PS template, it is reasonable to give the ring pattern for the 2D WAXD pattern due to the acquired diffraction results from polycrystalline materials with grain boundaries. To acquire diffraction within the forming monograin, selected-area electron diffraction (SAED) experiment was carried out. As shown in Figure 5a, the TEM image of the microsection of PS/ZIF-67 appears as diamond-structured ZIF-67 with different projections. As shown in Figure 5b, by selecting one of the monograins for electron diffraction, a single-crystal-like diffraction pattern along the [111] zone axis can be identified. These results reflect that the nature of the mesoporous ZIF-67 fabricated by templated synthesis gives rise to the growth of single-crystal ZIF-67 from the template with diamond-structured monograin (see below for details).

**Figure 3:**
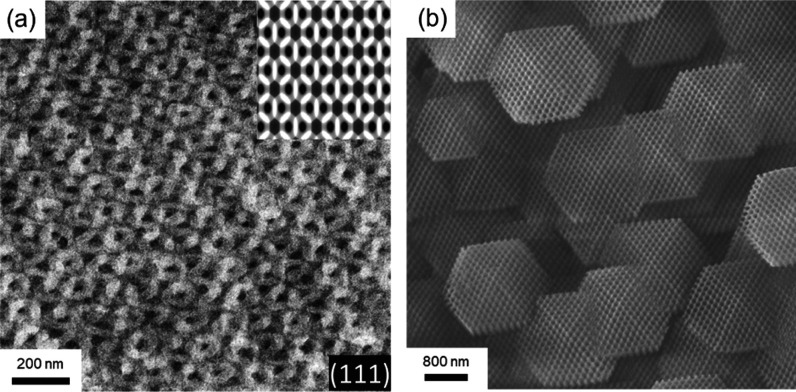
(a) TEM micrograph of PS/ZIF-67 nanohybrids showing the projection of a double diamond phase along the [111] direction. (Inset) Corresponding image from simulation based on the double diamond phase. (b) FESEM image of mesoporous ZIF-67 fabricated by the templated coordination-driven self-assembly reaction of ZIF-67 after the removal of the PS template.

**Figure 4:**
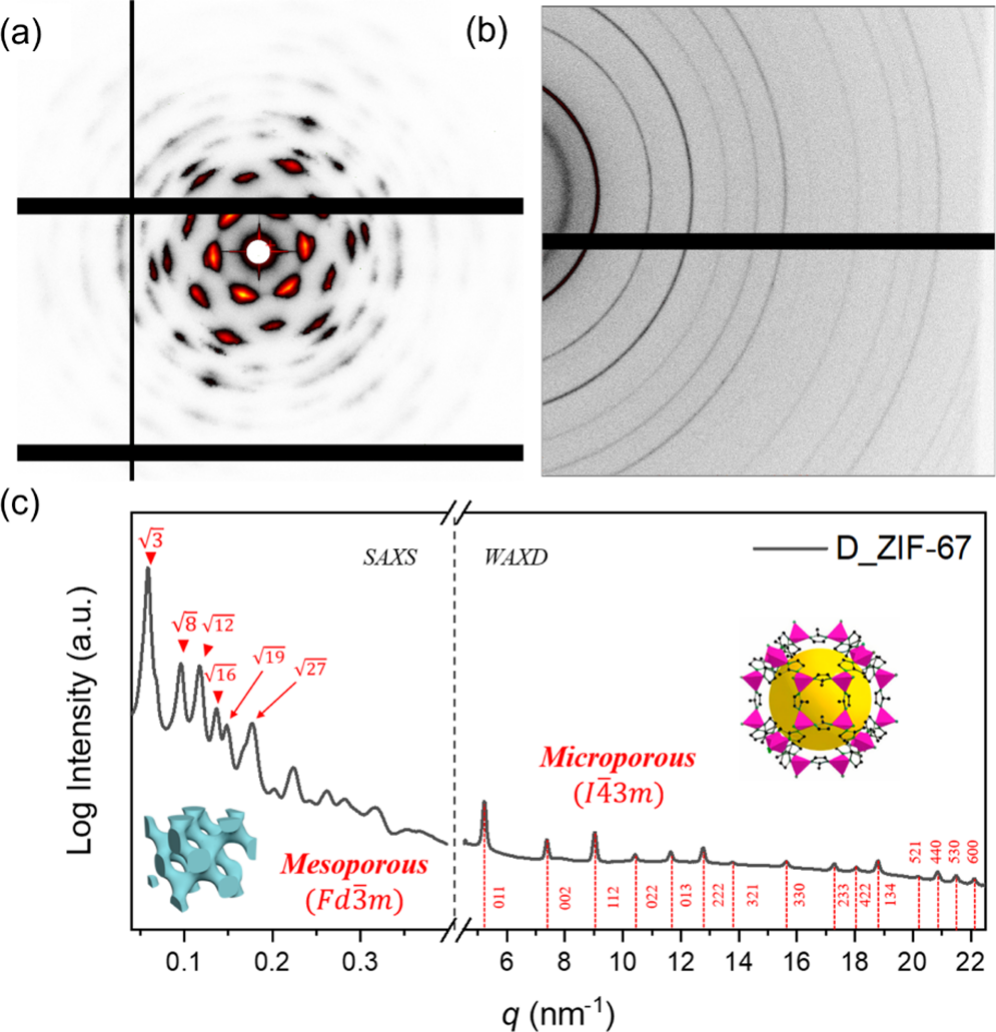
(a) 2D SAXS pattern of diamond-structured ZIF-67, unveiling the reflections with single diamond texture from a double diamond template; (b) 2D WAXD pattern of mesoporous ZIF-67 with high crystallinity; (c) corresponding 1D X-ray profile of the diamond-structured ZIF-67 with high crystallinity.

**Figure 5:**
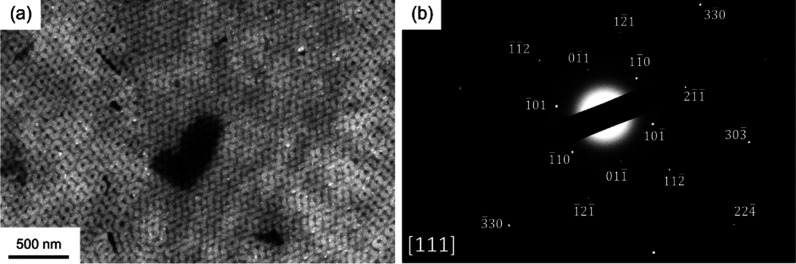
(a) TEM micrograph of diamond-structured PS/ZIF-67 nanohybrids. (b) Corresponding SAED pattern from the monograin with diamond-structured ZIF-67 along the [111] zone axis.

The inherent morphology of the ZIF-67 single crystal exhibits a typical rhombic dodecahedral structure characterized by 12 congruent rhombic faces. However, the morphology of the mesoporous ZIF-67 single crystal fabricated by templated synthesis gives a cuboctahedron instead of the typical rhombic dodecahedron, which is a convex polyhedron with eight triangular faces and six square faces.^50^ Figure S2 illustrates this morphology change, while Figure S3 provides schematic representations of both polyhedral shapes. For an intrinsic crystal growth of the ZIF-67 single crystal, the growth initiates with a cube shape, showcasing six {100} facets. These cubes then transform into a truncated rhombic dodecahedron with six {100} and twelve {110} facets. Ultimately, it evolves into a thermodynamically stable rhombic dodecahedron, exposing only the twelve {110} facets due to the consideration of the growth rate at which the morphological evolution from a cube to a rhombic dodecahedron occurs because the growth rate of ZIF-67 crystals is slower in the (100) direction than in the (110) direction. This phenomenon can be attributed to the varying exposed crystallographic planes of the crystal during morphological evolution. Figure S4 summarizes this intrinsic growth process, and Figure S5 illustrates how directional constraints under confinement may alter the typical evolution path. As shown in Figure 6, the {100} and {211} planes in the ZIF-67 crystal exhibit the highest Co-2-MeIm linkage density, while the {110} and {111} planes lack them. This indicates that the exposure of {110} and {111} should be the most thermodynamically stable conditions. Facets with a lower linker density may have fewer unsaturated coordination sites or metal centers. These sites are typically high-energy sites because they are not fully coordinated. With fewer such sites, the overall surface tension can be reduced, leading to lower surface energy. This greatly explains why the shape of the ZIF-67 single crystal ultimately evolves into a rhombic dodecahedron, which exposes 12 {110} facets.^51^ As mentioned previously, the crystal growth of ZIF-67 starts from a cubic formation, with six {100} planes exposed. The possibility of shape transitions from a cube to a cuboctahedron occurs only if the growth rate in the (111) direction is slower than that in the (100) direction. The observed phenomenon raises the question of its applicability to the situations under confinement (i.e., templated coordination-driven self-assembly reaction). Notably, the single crystal grown in this study is indeed confined in a network nanochannel and developed with a single diamond texture in nanoscale. The diamond lattice can be conceptualized as an FCC-like structure (FCC structure with an extra atom placed at 1/4a_1_ + 1/4a_2_ + 1/4a_3_ from each of the FCC atoms). The template for this FCC-like structure inherently has eight corners. Consequently, when the crystal grows, the growth rate at these eight corners, namely in the ⟨111⟩ direction, is physically constrained by the template. This physical hindrance leads to the formation of thermodynamically unstable (100) crystal faces. Note that, with the increase of thermodynamically unstable crystal faces, it is possible to enhance the catalytic efficiency due to their higher surface energy and increased density of Lewis acid active sites. These properties will be able to promote effective interactions and reactions as the atoms on these faces can be much more reactive, thus providing a larger number of reactive sites for catalytic activity.

**Figure 6:**
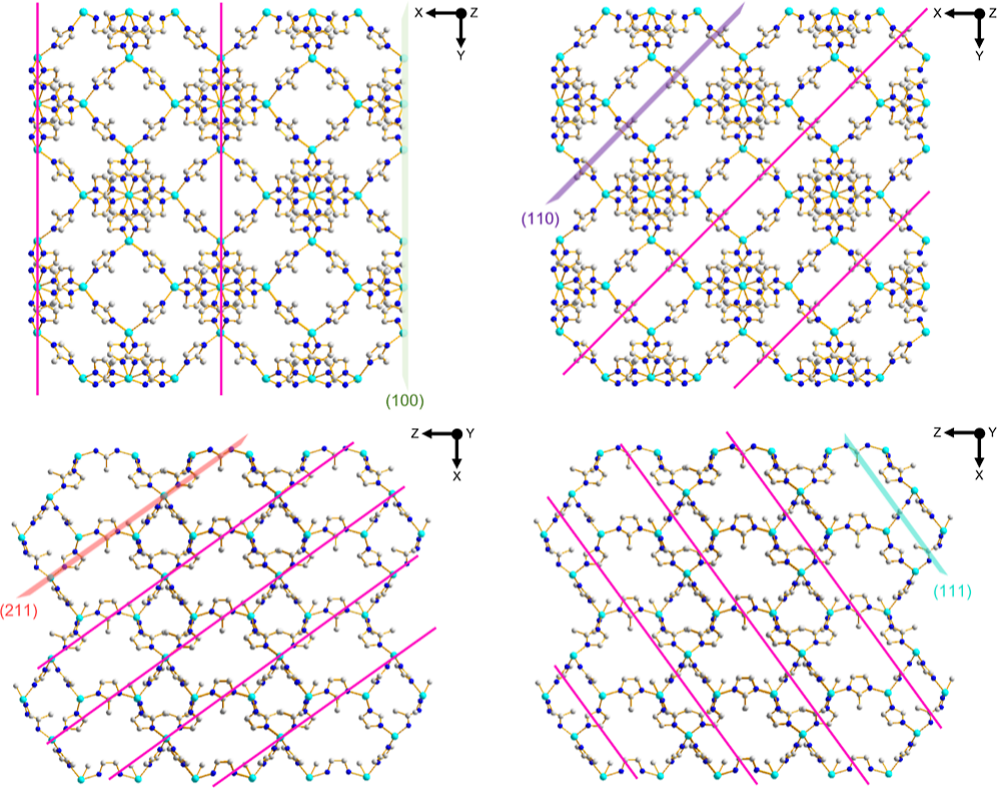
Schematic illustration of the crystal structure of ZIF-67 along different directions. The Miller index associates with the ZIF-67 single crystal. The (100) and (211) planes contain the highest density of Co^2+^- 2-MeIm linkage. Cobalt ions are represented as light blue spheres and carbon and nitrogen atoms as white and dark blue, respectively.

To further confirm the proposed mechanism, a template with a gyroid structure was used for comparison. The gyroid form factor can be partly considered an effective sphere because it simplifies the complex tripod structure with varying thicknesses into a manageable model. The smooth curvature and isotropic properties of the sphere capture the essential characteristics of the form factor of gyroid, giving the sphere a reasonable approximation for the building block of the gyroid. In contrast to the ZIF-67 single crystal fabricated from a diamond-structured template (Figure 7a,b), the ZIF-67 single crystal fabricated from a gyroid-structured template tends to resemble the texture with a sphere-like polyhedron (Figure 7c,d). By applying the proposed mechanism that was mentioned earlier, the gyroid structure is a mathematically defined minimal surface characterized by its continuous, triply periodic form with no self-intersections and no straight segments, making it challenging to simplify into a basic form. This complexity, however, underscores the idea that the template can indeed give rise to control of the crystal growth shape due to physical constraints, subsequently affecting its crystal facets.

**Figure 7:**
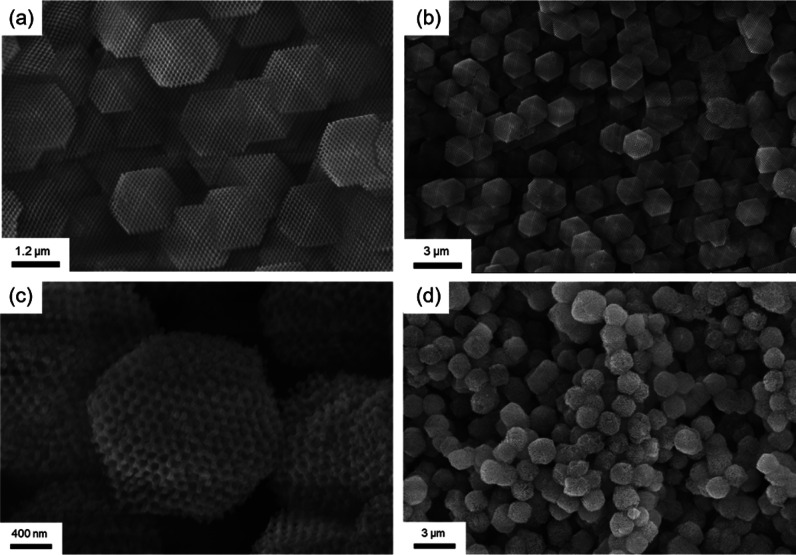
FESEM images of ZIF-67 single crystals fabricated by templates with different structures. (a,b) Single diamond-structured ZIF-67 single crystals; (c,d) single gyroid-structured ZIF-67 single crystals.

Interestingly, it has been observed that the size of the ZIF-67 single crystal can be manipulated by merely adjusting the concentration of the precursors. Based on the crystallization processes from the templated synthesis, it is intuitive to expect that the number of nucleation sites inversely affects the size of the resultant crystals. Nucleation sites can serve as initiation points for crystal formation. When numerous nucleation sites are present, multiple crystals form concurrently, giving rise to competition for the available solutes to carry out the coordination-driven self-assembly reaction; as a result, it is reasonable to expect the growth of the crystal with a smaller size. Conversely, with fewer nucleation sites, crystals have more solutes and space to expand, resulting in fewer but larger crystals. Four different represented concentrations of the metal ion precursor in methanol, namely 4 M, 2.75 M, 1.38 M, and 0.68 M, were utilized to monitor the structural evolution of the products on a Co­(NO_3_)_2_·6H_2_O basis while holding the molar ratio of 2-MeIM/Co­(NO_3_)_2_ at 8. Figure 8 shows the diamond-structured ZIF-67 single crystals fabricated using different concentrations of the precursors. As observed, the particle size of the ZIF-67 single crystal becomes larger and larger as the concentration gradually decreases. Figure S6 provides additional FESEM images at different precursor concentrations, confirming that higher concentrations lead to smaller crystal sizes. Furthermore, FESEM images consistently reveal the presence of highly ordered single diamond structure with specific facet texture for the ZIF-67 single crystal fabricated regardless of the concentration, which further evidence the suggested mechanism for the confined crystallization. The diamond texture can be well preserved, and even the size of ZIF-67 exhibits a single diamond texture. This phenomenon, where a single diamond texture is formed from a double diamond-forming template, can be attributed to the low nucleation density of the initial ZIF-67 nuclei. With a decrease in the nucleation density, single diamond-structured ZIF-67 can be fabricated due to the annihilation and growth of ZIF-67 crystals simultaneously from the neighboring nanochannels. Consequently, single diamond-structured ZIF-67 single crystals with controlled particle sizes can be obtained by tuning the concentration of precursor in methanol through the nucleation and growth processes. Figure S7 shows that the size distributions of these crystals are narrow under various concentrations of the precursor, suggesting the presence of an Ostwald ripening mechanism during growth. To further demonstrate the control of the size for the mesoporous ZIF-67 single crystal, the average sizes of the particles were calculated. Figure 9 shows the relationship between the concentration of the precursors and the particle size. The particle size histograms in Figure S8 reveal a Gaussian distribution, confirming the excellent uniformity of particle sizes fabricated at different precursor concentrations. By controlling the rate at which new crystal nuclei can be tuned by the concentration of the precursor, the overall number of nuclei can be regulated. A lower nucleation rate generally leads to fewer nuclei, which allows for the growth of larger crystals. Conversely, a higher nucleation rate results in a larger number of nuclei and thus smaller size of the crystal grown.

**Figure 8:**
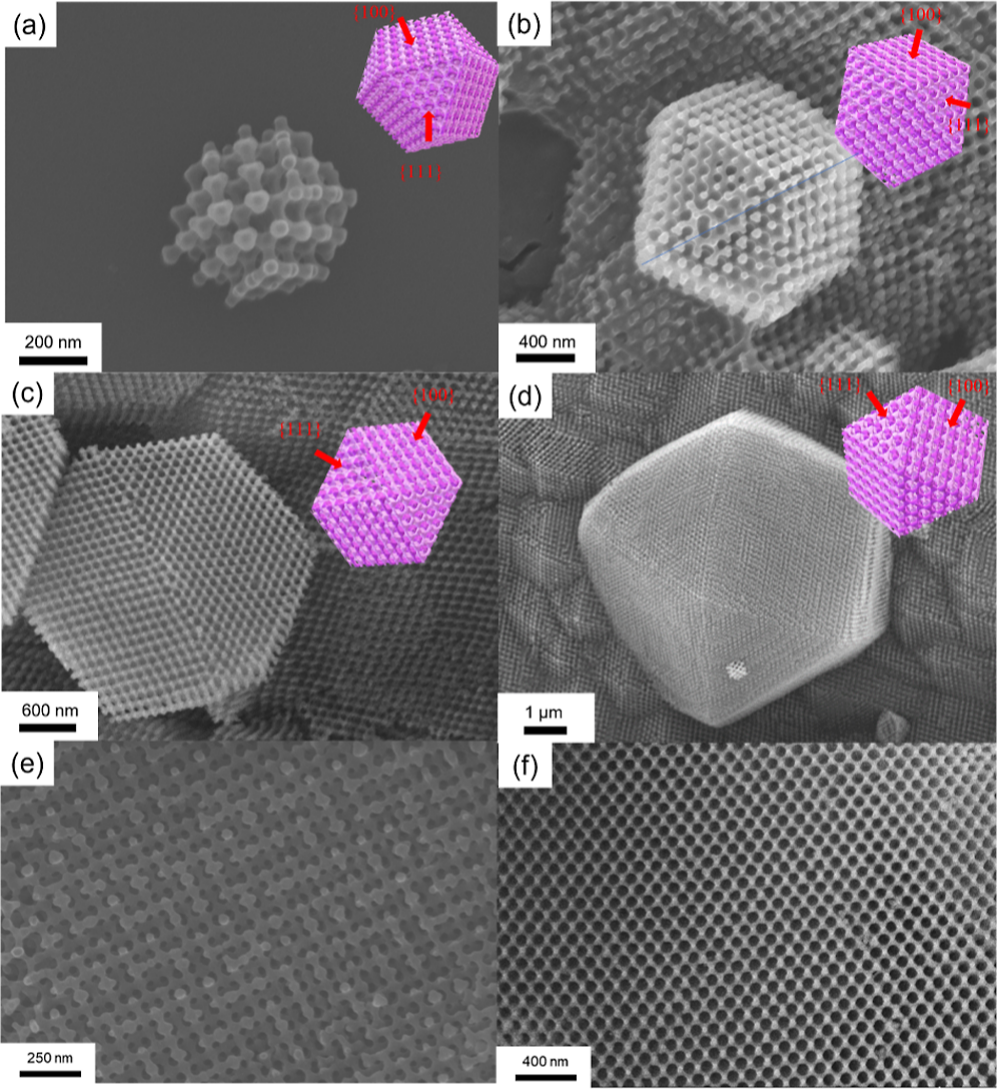
FESEM images of well-ordered nanonetwork ZIF-67 fabricated under different concentrations of the metal ion precursor in methanol. (a) 4 M; (b) 2.75 M; (c) 1.38 M; (d) 0.68 M. (Insets) The corresponding images of cuboctahedron taken from different directions. (e,f) Enlarged FESEM micrographs of (d) from [114] and [111] directions, respectively, showing single diamond texture possessing a network structure with nanometer strut.

**Figure 9:**
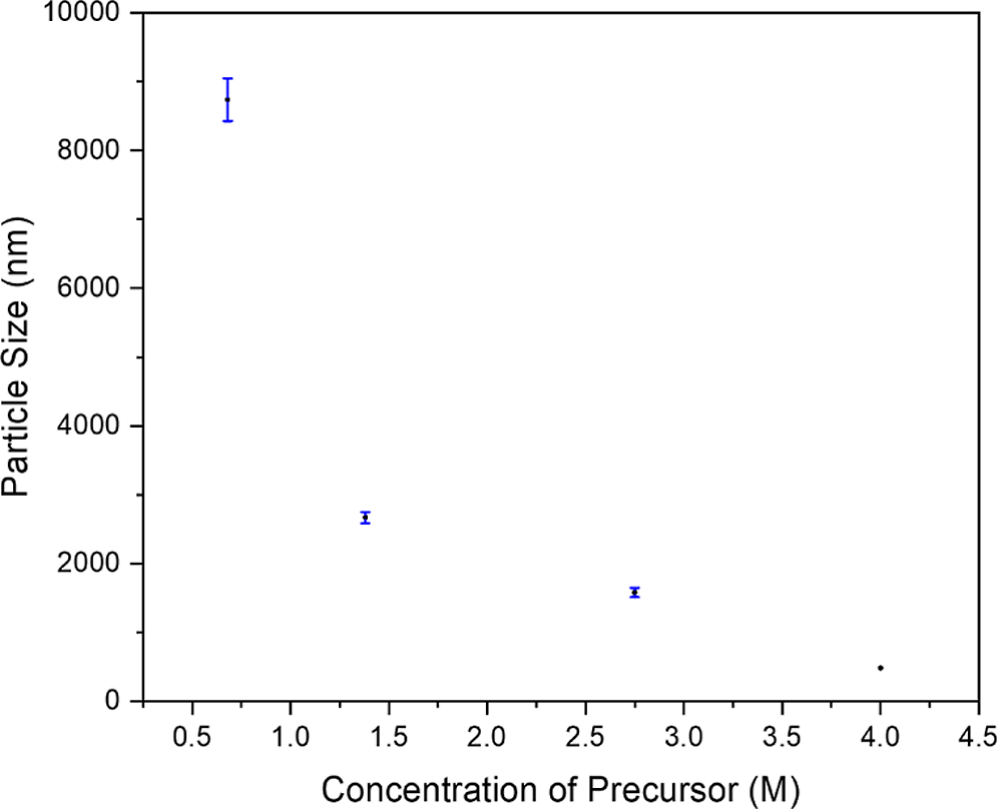
Relationship between the concentration of metal ion precursors in methanol (4, 2.75, 1.38, and 0.68 M) and the corresponding particle sizes of diamond-structured ZIF-67 single crystals (approximately 480 nm, 1580 nm, 2670 nm, and 8740 nm, respectively). Error bars represent standard deviation.

To examine the mechanical properties of the nanonetwork ZIF-67 single crystals fabricated, nanoindentation tests were conducted to compare the intrinsic ZIF-67 crystal with the fabricated nanonetwork ZIF-67 single crystal. As shown in Figure 10a, intrinsic ZIF-67 possesses the typical load–displacement curves with the use of the spherical indenter with a diameter of 2 μm under the maximum loadings of 500, 1000, and 1500 μN. Note that intrinsic ZIF-67 can be considered as an absolute elastic response with the least displacement after unloading that is attributed to the intrinsic brittleness with minimal energy dissipation. For instance, it is approximately 320 nm after the removal of the loading of 1500 μN. In contrast to intrinsic ZIF-67, nanonetwork ZIF-67 (Figure 10b) shows larger displacement after unloading; for instance, it is approximately 580 nm after the removal of the load of 1500 μN. These results suggest that the well-ordered nanonetwork ZIF-67 single crystal demonstrates improved energy dissipation, attributed to the deliberate structuring effect on mechanical properties. Note that the energy dissipation capability can be evaluated by integrating the area under the load–displacement curve. As calculated by integrating the area of the closed-loop at a maximum loading of 1500 μN, the nanonetwork ZIF-67 shows a significant enhancement of the value of 0.56 nJ as compared to the intrinsic ZIF-67 value of 0.27 nJ. Moreover, an energy dissipation index, which is the relative contribution of energy dissipation (*W*_p_) to the total absorbed energy (*W*_t_ = *W*_p_ + *W*_e_), where *W*_e_ is the elastically stored energy, was used to further confirm the enhanced energy dissipation of the nanonetwork ZIF-67.

**Figure 10:**
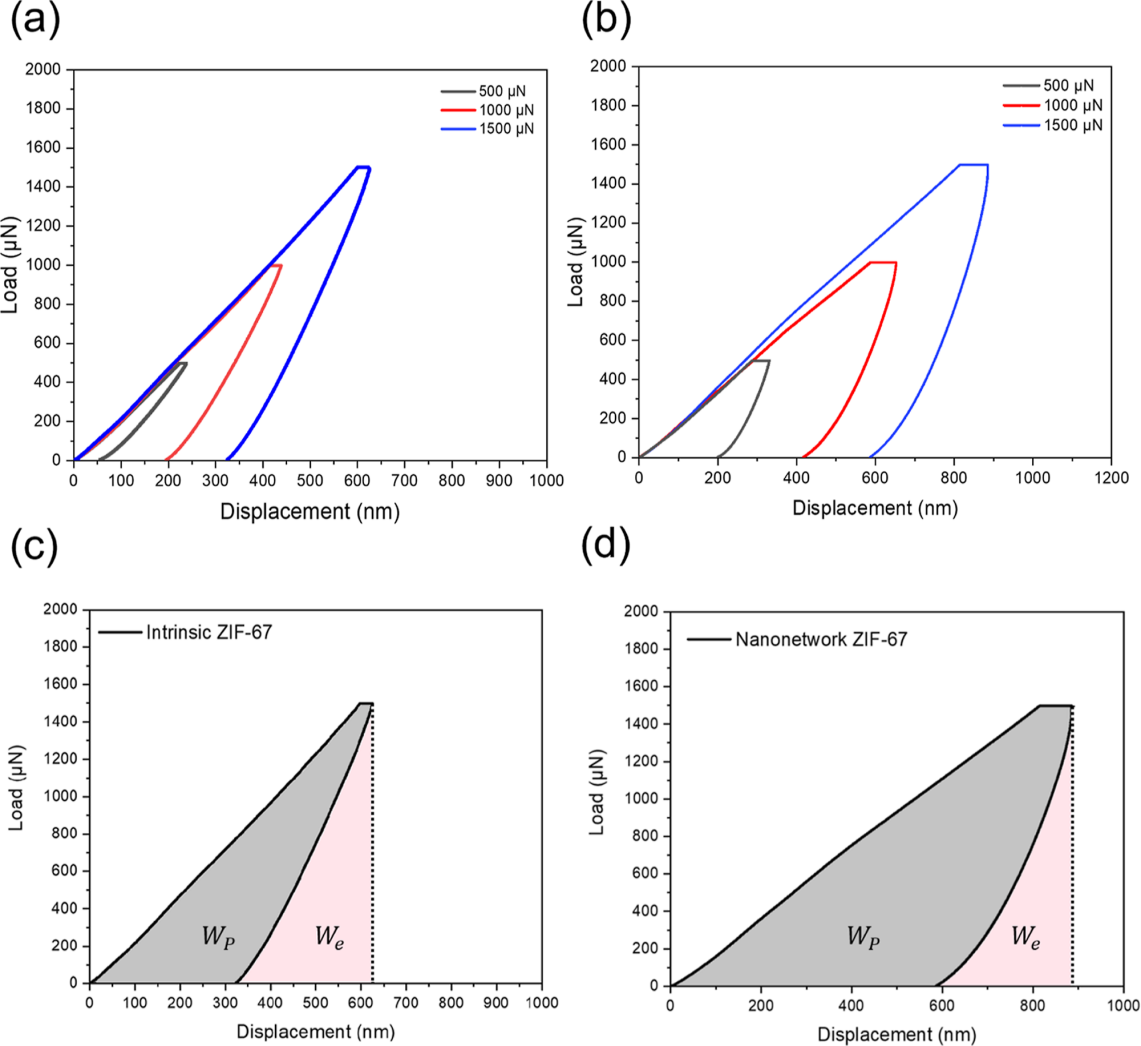
Load–displacement curves of (a) intrinsic ZIF-67 single crystals and (b) diamond-structured ZIF-67 nanonetworks at three different peak loads (500, 1000, and 1500 μN). Energy absorption by plastic deformation during nanoindentation at 1500 μN for (c) intrinsic ZIF-67 and (d) diamond-structured ZIF-67 nanonetworks, showing plastic energy (*W*_p_, gray) and elastic energy (*W*_e_, pink). The total contact energy (*W*_t_ = *W*_p_ + *W*_e_) was calculated to be 0.47 nJ and 0.71 nJ, with the corresponding energy dissipation ratios (*W*_p_/*W*_t_) of 57.4% and 78.8%, respectively.

The intrinsic ZIF-67 crystal exhibits a total contact energy (*W*_t_) of 0.47 nJ under a peak load of 1500 μN, with the calculated energy dissipation index (*W*_p_/*W*_t_) of 57.4%, as illustrated in Figure 10c. In contrast, the diamond-structured ZIF-67 nanonetwork shows a significantly higher *W*_t_ value of 0.71 nJ and a dissipation index of 78.8%, as shown in Figure 10d. This substantial increase in energy absorption through plastic deformation highlights the mechanical resilience imparted by the interconnected periodic architecture of the nanonetwork MOF. The reliability of this comparison can be supported by multicycle nanoindentation tests (Figure S9), which show consistent load–displacement curves for intrinsic ZIF-67 under different loads, indicating excellent repeatability of mechanical measurements. Such enhancement can be attributed to the deliberate structuring in nanoscale, which enables efficient stress distribution and dissipation throughout the framework.^14,52^

In conclusion, this study successfully demonstrates the fabrication of nanonetwork ZIF-67 single crystals with mesoscale network structures, highlighting their potential for multifunctional applications. Using the controlled self-assembly of lamellae-forming PS-*b*-PDMS, a network-structured template was developed, enabling the templated coordination-driven reaction of mesoporous ZIF-67 single crystals. Characterization through SAXS, wide-angle diffraction, and electron diffraction revealed the formation of single-network ZIF-67 single crystals, attributed to low nuclei density and the physical constraints imposed by the template. These constraints stabilized higher-reactivity crystallographic planes while preventing the intersection of interpenetrating networks, underscoring the critical role of the template design in controlling crystal growth and morphology. Furthermore, the nanonetwork periodic structuring significantly enhances the mechanical properties of mesoporous ZIF-67 due to the characteristic of mechanical metamaterial, as evidenced by nanoindentation tests showing a brittle-to-ductile transition, thus increasing the energy dissipation capabilities. The energy dissipation index improved from 57.4% to 78.8%, corresponding to a transformative impact on plastic deformation and energy absorption. Overall, this work underscores the importance of deliberate nanonetwork structuring in tailoring the mechanical functionality of a MOF and establishes a robust platform for designing advanced materials with exceptional energy absorption and impact resistance for high-performance applications.

## Supplementary Material



## Notes

Sample preparation and experimental details (PDF)
