# Supplementary material for: Mesoporous Metal–Organic Framework from Templated Synthesis as Mechanical Metamaterials
Source: J Am Chem Soc. 2025 Jun 29;147(27):23608–16. doi: 10.1021/jacs.5c04214 (PMC12257539; doi:10.1021/jacs.5c04214)
Supplement: Supplementary file 1 [file ja5c04214_si_001.pdf]

Supporting Information for

Mesoporous Metal-Organic Framework from Templated Synthesis as Mechanical Metamaterials

Ting-Wei Liang,[# a] *Chien Chen,* [# a] *Shinpei Kusaka,* [b] *Suhail K. Siddique,*[a,c] *Cheng-Yen Chang,*[a] *Ryotaro Matsuda,*[b] *Rong-Ming Ho**[a]

[#] Equal contribution

[a] Department of Chemical Engineering, National Tsing Hua University No. 101, Section 2, Kuang-Fu Road, Hsinchu, Taiwan 30013, R.O.C.

[b] Department of Materials Chemistry, Graduate School of Engineering, Nagoya University, Furo-cyo, Chikusa-ku, Nagoya 464-8603, Japan

[c] Department of Nuclear and Mechanical Engineering, Khalifa University of Science and Technology. Abu Dhabi, UAE.

Materials

Cobalt(II) nitrate hexahydrate ($Co(NO_3)_2 \cdot 6H_2O$, 98% ,Sigma-Aldrich), 2-methylimidazole (99%, Sigma-Aldrich), Chloroform (J.T. Baker), dichloromethane (J.T. Baker), hydrofluoric acid (HF, Sigma-Aldrich), methanol (Honeywell).

Method

Synthesis of the PS-*b*-PDMS Diblock Copolymer

The synthesis of the lamellae-forming PS-*b*-PDMS was conducted *via* anionic polymerization, as previously reported in our publications.[1, 2] We initially prepared a sample with polystyrene (PS) and polydimethylsiloxane (PDMS) molecular weights of 51,000 g/mol and 35,000 g/mol, respectively, resulting in a PDMS block volume fraction of 0.42. Additionally, we synthesized another sample using PS and PDMS with molecular weights of 47,000 g/mol and 33,000 g/mol, respectively, which achieved a PDMS block volume fraction of 0.46. This method facilitated the precise fabrication of copolymers with varied compositions. **Table 1** presents the PS-*b*-PDMS samples used in this study.

Sample	M_n^{PS} (kgmol^{-1})	M_n^{PDMS} (kgmol^{-1})	$Đ_M$	f_{PDMS}^{v}
PS-*b*-PDMS	51.0	35.0	1.02	0.42
PS-*b*-PDMS	47.0	33.0	1.04	0.46

Table S1 Characterization of the synthesized PS-*b*-PDMS.

Self-assembled PS-*b*-PDMS from Solution Casting

In order to fabricate nanonetwork materials through BCP templated synthesis, it is essential to have a straightforward method for preparing porous polymer templates with well-defined continuous nanochannels. Achieving network morphology through the

self-assembly of BCPs can be challenging due to the limited window of network phases in the typical phase diagram of diblock copolymers. As demonstrated by our laboratory, it is possible to kinetically trap metastable network phases during the self-assembly process of PS-*b*-PDMS through a lamellae-forming PS-*b*-PDMS by using a selective solvent for casting. As shown in **Figure S1a** alternating dark and bright strips at which

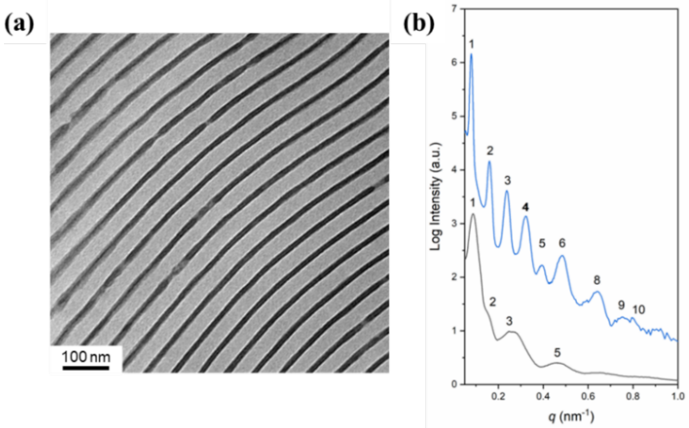

Figure S1. (a) TEM micrograph of self-assembled PS-*b*-PDMS after solution casting by cyclohexane. (b) Corresponding 1D SAXS profile after solution casting using cyclohexane (black line) and followed by thermal annealing at 180˚C for 24 hours (blue line).

PDMS microdomains appear dark and the PS matrix is the bright area due to the mass contrast from silicon-containing PDMS phase can be observed, suggesting the formation of lamellar phase with the use of cyclohexane (neutral solvent) for casting, as further evidenced by the 1D SAXS result with the reflections occurred at the relative q values of 1, 2, 3, 5 (**Figure S1b**). The solution-casted sample is subsequently thermally annealed at 180°C for 24 hours to eliminate distortions and enhance structural order. The resulting TEM projection confirms the formation of lamellar-structured PS-*b*-PDMS. The enhanced order post-annealing is substantiated by the expanded series of specific reflections at relative q-values of 1, 2, 3, 4, 5, 6 in the SAXS profile shown in **Figure S1b**.

Fabrication of Mesoporous Template

The fabrication process entailed immersing the diamond-structured PS-*b*-PDMS thin film in a hydrofluoric acid (HF) solution (HF/methanol, 1:4 by volume) for 7 days to selectively remove the PDMS block. The most crucial step is to ensure that the bulk sample is fully immersed in the mixed HF/methanol solution. After the HF etching, the sample was rinsed in methanol for one hour to remove any residual HF solution. This process produced nanoporous PS templates with through-pore nanochannels. Finally, the HF-etched PS-*b*-PDMS templates were dried under vacuum at ambient temperature for one hour.

Templated Synthesis of Nanonetwork ZIF-67 Single Crystal

Either diamond-structured or gyroid-structured ZIF-67 single crystal is synthesized through the same approach. For the pore-filling of ZIF-67, the conventional solvothermal method was employed to self-assemble the ZIF-67 precursor through a coordination-driven self-assembly reaction, resulting in the formation of a ZIF-67 single crystal within the mesoporous PS template. The nanonetwork ZIF-67 sample was prepared following a previously reported literature with slight modifications.68 Initially, 2g of $Co(NO_3)_2{\cdot}6H_2O$ and 5g of 2-methylimidazole were both dissolved in 5 ml of methanol, respectively. Afterward, the mesoporous PS template was immersed in the $Co(NO_3)_2{\cdot}6H_2O$ solution for one day to allow the precursor solution to thoroughly diffuse into the PS template. Subsequently, with vigorous stirring, the solution of 2-methylimidazole was slowly added to the $Co(NO_3)_2{\cdot}6H_2O$ solution, resulting in the formation of purple precipitates as the two solutions mixed. However, with the above-mentioned procedure, ZIF-67 crystals form randomly in the solution instead of forming inside the PS template. Additionally, the PS template exhibited cracking when subjected to vigorous stirring in the solution. Hence, to prevent rapid reactions of the precursors,

which can block nanochannels by forming large nanocrystals and hinder pore filling, the precursor solution was impregnated into the template one after another. The mesoporous PS template was first soaked in the metal ion precursor solution. Next, the fully impregnated template was transferred to the organic ligand precursor solution to ensure that the coordination-driven self-assembly reaction mostly occurs within the nanochannels of the template. After that, the solution is stirred for 10 minutes to ensure the mixture of the molecules. The solution is placed at ambient temperature for more than 12 hrs. The purple bulk was taken out and washed repeatedly with methanol to remove the residual precursor and the unfilled ZIF-67 crystal. Finally, the sample is vacuum-dried overnight at 60°C to remove the guest molecules.

Removal of PS Template

The complete removal of the PS template was necessary to achieve nanonetwork-structured materials with a mesoporous texture. Metal-Organic frameworks are well known for their exceptional chemical stability. Hence, for ZIF-67/PS composite samples, a straightforward method is used for PS template removal. In this study, the nanonetwork-structured ZIF-67/PS bulks were immersed in the chloroform for 15 minutes. After being taken out from the chloroform, the nanonetwork ZIF-67 is rinsed with chloroform again three times to eliminate the residue PS template and any unreacted ZIF-67 precursors. Finally, the nanonetwork ZIF-67 is vacuum-dried overnight at 60°C for complete removal of chloroform.

Small-angle X-ray Scattering (SAXS)

Small-angle X-ray scattering (SAXS) experiments were conducted at the BL23A1 synchrotron X-ray beamline located at the National Synchrotron Radiation Research Center (NSRRC). The X-ray beam had a wavelength of 0.155 nm. We utilized a MAR CCD X-ray detector (MAR USA) for the collection of two-dimensional (2D) SAXS

patterns. To obtain one-dimensional (1D) linear profiles, we performed an integration of the 2D pattern. The calibration of the SAXS pattern's scattering angle was accomplished using silver behenate, setting the first order scattering vector q^* ($q^* = 4\lambda^{-1} \sin\theta$, where 2θ is the scattering angle) at 1.076 nm^{-1}. Scattering intensity profiles were generated, displaying the scattering intensity (I) about the scattering vector (q), where $q = (4\pi/ \lambda) \sin(\theta/2)$, with θ representing the scattering angle. All experiments were conducted at room temperature.

Transmission Electron Microscopy (TEM)

Bright-field transmission electron microscopy (TEM) images were obtained using the mass thickness contrast with a JEOL JEM-2100 LaB6 transmission electron microscope (at an accelerating voltage of 200 kV). The bulk samples were sectioned at room temperature by Leica Ultra-microtome. Then, the microsections were collected on copper grids (100 mesh).

Nanoindentation Measurements

Hysitron TI950 triboindenter (Hysitron Inc.) was used to perform the nanoindentation tests fitted with a spherical indenter of tip diameter of approximately 2 μm; the indentation measurements were conducted in bulk samples with 10 μm thickness with silicon wafer as a substrate at room temperature. The load-displacement curve with a maximum load of 500, 1000 and 1500 μN applied at the same rate of loading and unloading (60 μN/sec) was acquired. In the nanoindentation tests, the load-displacement data were recorded continuously while the tip was driven into the composite materials, and then smoothly removed. The load-displacement (L-D) curves were then used to calculate the mechanical energy dissipation of the fabricated materials.

Morphological Evolution of ZIF-67 Single Crystal in Confined Space.

As illustrated in **Figure S2a**, the intrinsic morphology of the ZIF-67 crystal exhibits a typical rhombic dodecahedron structure. However, the morphology of nanonetwork

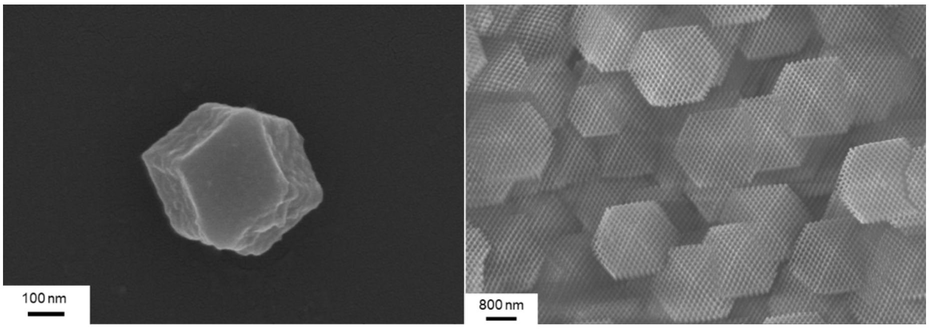

Figure S2. FESEM image of (a)intrinsic ZIF-67 single crystal with rhombic dodecahedron morphology. (b) diamond-structured ZIF -67 single crystal exhibits a cuboctahedral morphology.

ZIF-67 fabricated by templated synthesis varies from a typical rhombic dodecahedron to a cuboctahedron (**Figure S2b**). The cuboctahedron is a convex polyhedron with 8 triangular faces and 6 square faces. This means it combines characteristics of both a cube (with its square faces) and an octahedron (with its triangular faces). In contrast, the rhombic dodecahedron is a convex polyhedron with 12 congruent rhombic faces. **Figure S3** provides images of the corresponding shape of (a) rhombic dodecahedron and (b) cuboctahedron, respectively.

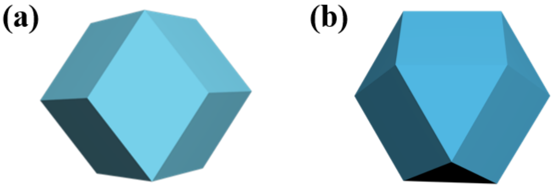

Figure S3. (a) 2D representation showcasing the unique shape of a rhombic dodecahedron; (b) 2D representation showcasing the unique shape of a Cuboctahedron.

To understand the mechanism of morphological changes when ZIF-67 particles crystallize in a template, it's crucial to grasp the intrinsic crystal growth mechanism of ZIF-67 itself. The forming mechanism of ZIF-67 single crystal has been well-investigated in the past few years. For intrinsic crystal growth of the ZIF-67 single crystal, the growth initiates with a cube truncated corners (truncated rhombic dodecahedron) (c and d) and rhombic shape, showcasing six {100} facets. These cubes then transform into truncated rhombic dodecahedron with six {100} and twelve {110} facets. Ultimately, they evolve into the thermodynamically stable rhombic dodecahedron, exposing only the twelve {110} facets (**Figure S4**).[3, 4]

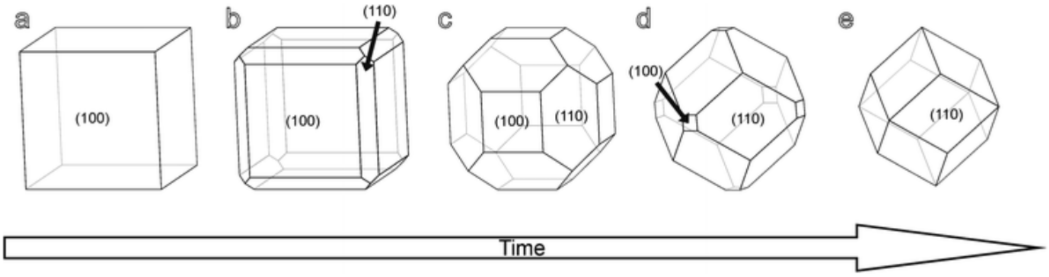

Figure S4. Illustration of the crystal morphology evolution with time: cube (a), cube with truncated edges (b), rhombic dodecahedron with dodecahedron (e). Miller indices are given only for one representative face out of the different sets of symmetry-equivalent faces (crystals forms).[4]

As mentioned previously, the crystal growth of ZIF-67 starts from a cubic formation with six {100} plane exposed. The possibility of shape transitions from a cube to a cuboctahedron occurs only if the growth rate in the (111) direction is slower than that in the (100) direction (**Figure S5**). The observed phenomenon raises the question of its applicability to situations under confinement (*i.e.*, templated coordination-driven self-assembly reaction). Notably, the single crystal grown in this study is indeed

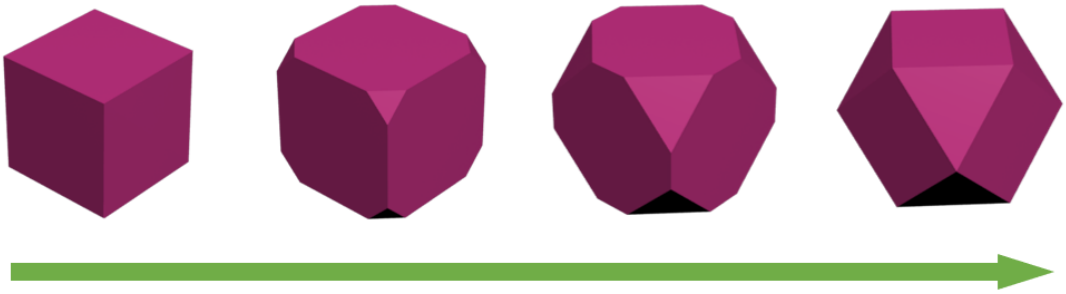

Figure S5. Illustration of the change in crystal morphology from cubic to cuboctahedron.

in a network nanochannel and developed with a single diamond texture in nanoscale. The diamond lattice can be conceptualized as an FCC-like structure (a FCC structure with an extra atom placed at $1/4a_1+1/4a_2+1/4a_3$ from each of the FCC atoms). The template for this FCC-like structure inherently has eight corners. Consequently, when the crystal grows, the growth rate at these eight corners, namely in the <111> direction, is physically constrained by the template. This physical hindrance leads to the formation of thermodynamically unstable (100) crystal faces. The induced thermodynamically unstable crystal faces are expected to enhance catalytic efficiency due to their higher surface energy and increased density of Lewis acid active sites. These properties promote more effective interactions and reactions, as the atoms on these faces are more reactive and provide more sites for catalytic activity.

Controlled Particle Size of Mesoporous ZIF-67 Single Crystal.

Figure S6a. shows the FESEM images of gyroid-structured ZIF-67 single crystals fabricated by different concentrations of precursor. Consistently, the particle size of the mesoporous ZIF-67 single crystal can be readily controlled by tuning the concentration of the precursors. Moreover, the variation trend in **Figure S6b.** experiences a level off with further increasing the precursor concentration to reach small particle size possibly due to the overlapping of the growing size from high nucleation. There is also an

important issue with respect to the controlled growth for the particle size of the mesoporous ZIF-67 single crystal at which the formation of the MOF single crystals indeed gives rise to a narrow distribution for the growth size as shown in **Figure S7**. This can be attributed to the Ostwald Ripening process.[5, 6]

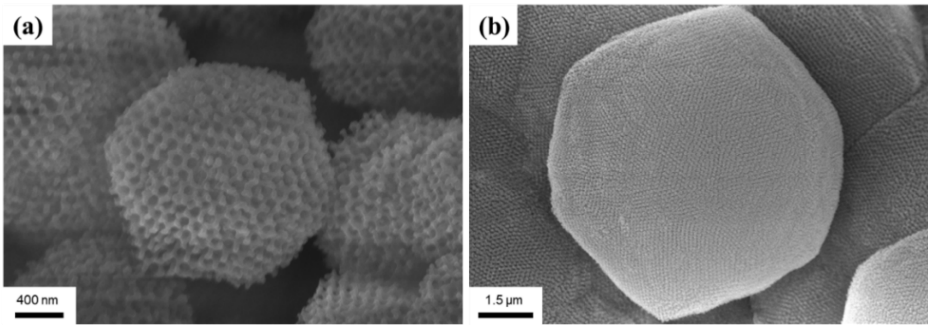

Figure S6. FESEM images of well-ordered nanonetwork ZIF-67 fabricated under different concentrations of precursor (a) 2.75M (b) 0.68M.

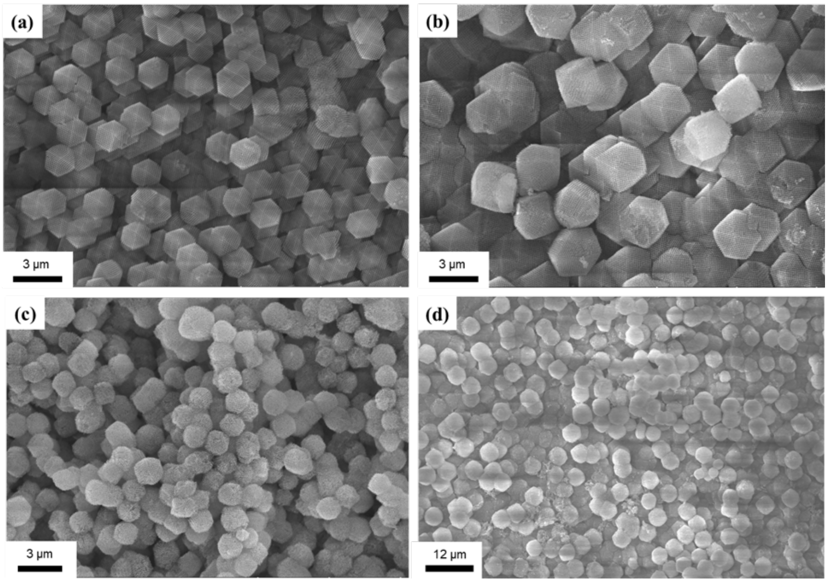

Figure S7. FESEM image show narrow size distribution of nanonetwork ZIF-67 particles with different size; (a) (5k magnification) of diamond-structured ZIF-67 particles with average size ≈ 1.58μm; (b) (5k magnification) of diamond-structured ZIF-67 particles with average size ≈ 2.7μm; (c) (4.5k magnification) of gyroid-structured ZIF-67 particles with average size ≈ 1.58μm; (d) (1.2k magnification) of gyroid-structured ZIF-67 particles with average size ≈ 8μm.

During this process, smaller crystals, which have higher surface energy and solubility, gradually dissolve. The dissolved material then re-deposits onto larger crystals, which are more thermodynamically stable and thus have lower surface energy. As the reaction progresses, the growth of larger crystals is preferentially promoted while the number of smaller crystals decreases. This results in a narrower size distribution as the system evolves towards fewer, uniformly larger crystals, minimizing the overall free energy and achieving a more stable configuration. The Ostwald Ripening process, therefore, ensures a uniform crystal size distribution, which is essential for the consistent performance of MOF materials in various applications.

To evaluate the morphological uniformity of diamond-structured ZIF-67 particles synthesized under different precursor concentrations, particle size distributions were measured and plotted as shown in **Figure S8**. Two representative samples synthesized at 2.75 M and 1.38 M precursor concentrations are presented in panels (a) and (b), respectively. The particles display average sizes of 1.58 μm (2.75 M) and 2.67 μm (1.38 M), and both histograms fit well to narrow Gaussian distributions. This indicates excellent size uniformity within each sample. The low standard deviation and absence of bimodal or broad size distribution further confirm the effectiveness of the template-confined growth in producing monodisperse ZIF-67 crystals. Such uniformity is essential for ensuring consistent mechanical performance across batches.

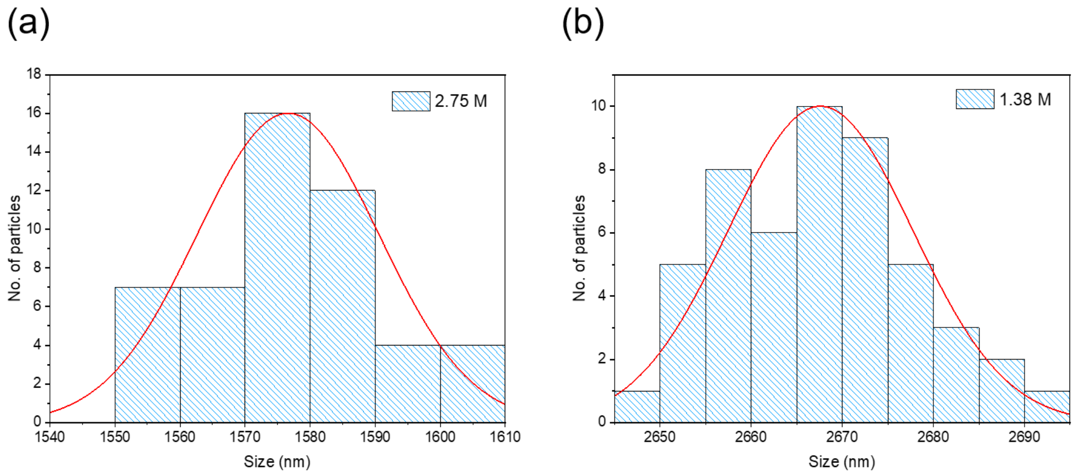

Figure S8. Representative particle size distributions of diamond-structured ZIF-67 particles: (a) particles with an average size of 2.67 μm (1.38 M); (b) particles with an average size of 1.58 μm (2.75 M). Both histograms exhibit narrow Gaussian distributions, indicating the high uniformity of particle size.

Reproducibility of Nanoindentation Test

To validate the reliability and repeatability of mechanical data, nanoindentation tests were systematically conducted on intrinsic ZIF-67 single crystals at three different peak loads: 500, 1000, and 1500 μN. As shown in **Figure S9**, each loading condition was repeated multiple times, and the corresponding load–displacement curves exhibit highly consistent profiles. The reproducibility can be evidenced by the overlapping curves within each load group, indicating minimal variations in contact stiffness and plastic deformation behavior. These results confirm that the mechanical response of ZIF-67 crystals should be stable under controlled test conditions; the measured hardness and reduced modulus are representative of the intrinsic material properties.

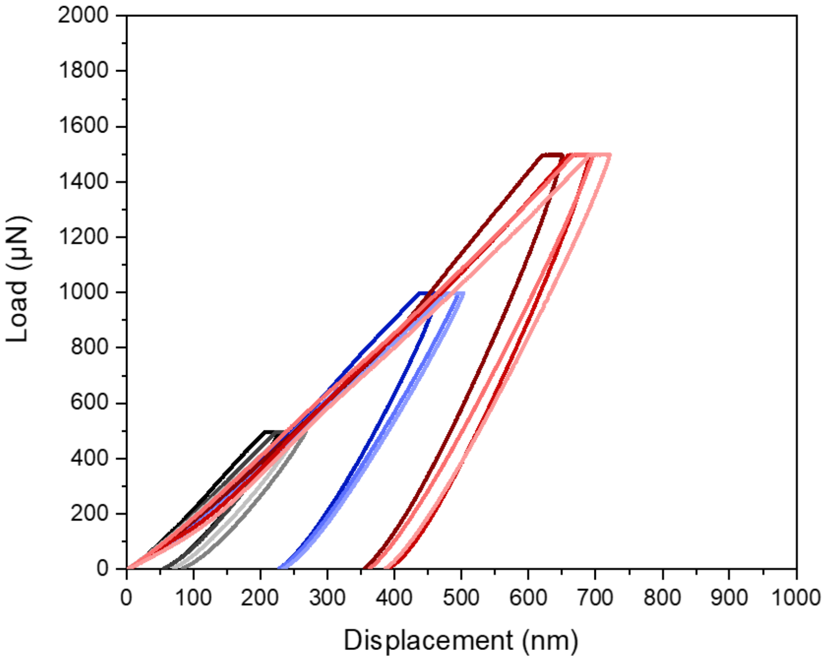

Figure S9. Represented load–displacement curves from nanoindentation multi-cycle tests on intrinsic ZIF-67 single crystals under three different peak loads (500, 1000, and 1500 μN).

References

[1] Lin, T. C.; Yang, K. C.; Georgopanos, P.; Avgeropoulos, A.; Ho, R. M. Gyroid structured nanoporous polymer monolith from PDMS-containing block copolymers for templated synthesis. *Polymer* **2017**, *126*, 360–367.

[2] Yang, K. C.; Yao, C. T.; Huang, L. Y.; Tsai, J. C.; Hung, W. S.; Hsueh, H. Y.; Ho, R. M. Single gyroid-structured metallic nanoporous spheres fabricated from double gyroid-forming block copolymers *via* templated electroless plating. *NPG Asia Mater* **2019**, *11*(1), 9.

[3] Cravillon, J.; Schröder, C. A.; Bux, H.; Rothkirch, A.; Caro, J.; Wiebcke, M. Formate modulated solvothermal synthesis of ZIF-8 investigated using time-resolved in situ X-ray diffraction and scanning electron microscopy. *CrystEngComm* **2012**, *14*

(2), 492-498.

[4] Cravillon, J.; Nayuk, R.; Springer, S.; Feldhoff, A.; Huber, K.; Wiebcke, M. Controlling Zeolitic Imidazolate Framework Nano- and Microcrystal Formation: Insight into Crystal Growth by Time-Resolved In Situ Static Light Scattering. *Chemistry of Materials* **2011**, *23* (8), 2130-2141.

[5] Park, J.; Joo, J.; Kwon, S. G.; Jang, Y.; Hyeon, T. Synthesis of Monodisperse Spherical Nanocrystals. *Angewandte Chemie International Edition* **2007**, *46* (25), 4630-4660.

[6] Peng, X.; Wickham, J.; Alivisatos, A. P. Kinetics of II-VI and III-V Colloidal Semiconductor Nanocrystal Growth: "Focusing" of Size Distributions. *Journal of the American Chemical Society* **1998**, *120* (21), 5343-5344.
